# Epigallocatechin Gallate Can Protect Mice From Acute Stress Induced by LPS While Stabilizing Gut Microbes and Serum Metabolites Levels

**DOI:** 10.3389/fimmu.2021.640305

**Published:** 2021-04-01

**Authors:** Yong Ma, Gang Liu, Muyang Tang, Jun Fang, Hongmei Jiang

**Affiliations:** College of Bioscience and Biotechnology, Hunan Agricultural University, Hunan Provincial Engineering Research Center of Applied Microbial Resources Development for Livestock and Poultry, Changsha, China

**Keywords:** Epigallocatechin gallate, LPS, serum metabolomics, acute injury, intestinal microbes

## Abstract

Epigallocatechin gallate (EGCG) has potent biological activity as well as strong antioxidant and anti-inflammatory effects. This study aims to explore the protective effect of EGCG on LPS-induced acute injury. We randomly divided 18 mice into three groups: CON, LPS, and EGCG-LPS. We gave the EGCG-LPS group gavage treatment with EGCG on day 8–15 and an intraperitoneal injection of LPS on day 16 to induce acute injury. The results showed that, compared with the LPS group, the bodyweight of the mice in the EGCG-LPS group increased significantly and effectively inhibited the morphological damage of the jejunum and liver. We measured liver tissue and found that the EGCG gavage treatment significantly inhibited the pro-inflammatory factors (*TNF-α, IL-1β, IL-6, MCP-1, MIP-2, IFN-γ*) and oxidation indicators (MPO, NO, ALT, and AST) levels increase. The microbiological results showed that the EGCG gavage treatment reshaped the disturbance done to the intestinal microbial community in the mice by LPS, reversed the changes in the abundance ratio of *Firmicutes*/*Bacteroidetes*, and significantly reduced the abundance of *Enterobacteriales*. Finally, the serum metabolomics results showed that, when compared with the LPS group, the gavage treatment of EGCG significantly increased the concentration of sphingomyelin (d17:1/17:0), sphingomyelin (d16:1/20:0), and significantly reduced the content of trans-Hexadec-2-enoyl carnitine, and so on. Therefore, we believe that EGCG can protect mice from acute stress induced by LPS while stabilizing gut microbes in general, improving the metabolism of sphingolipids, and inhibiting the content of harmful metabolites.

## Introduction

Lipopolysaccharide (LPS) is a major part of the cell wall of gram-negative bacteria, and it is composed of lipids and polysaccharides. A large amount of experimental data shows that LPS can cause systemic inflammatory response syndrome (SIRS) (1, 2). It is an endotoxin that can not only be secreted in the form of outer membrane vesicles (OMVs) but can also be released by forming micelles in the bacterial membrane (3). LPS is transported to the cytoplasm of immune cells in the form of OMVs, and inflammatory caspases, as the specific receptors of LPS, can activate the production of downstream pro-inflammatory factors (4). On the other hand, LPS contained in micelles is recognized by TLR4 under the combined action of binding proteins (5). Finally, the danger signal of the stress inflammation induced by LPS will reach the liver from the gastrointestinal tract, portal vein, or arterial blood circulation (6). LPS activates liver immune cells (Kupffer cells), thereby inducing the production of *TNF-α* and other inflammatory factors, which cause the liver to produce oxidative stress and inflammation (7). In an injured state, the liver will eliminate the damaged organelles through autophagy to reduce apoptosis, maintain the integrity of the tissue genome, and provide energy to ensure cell homeostasis (8). In addition, LPS and its receptor protein, *TLR4*, can induce small intestine damage and reduce its nutrient absorption capacity, thereby causing an imbalance of the intestinal flora and increasing the intestinal permeability. This process further promotes liver damage (9). This may be why the microbial metabolites after the disorder can promote liver inflammation and fibrosis (10). Therefore, for intestinal microbial homeostasis, we can use intestinal microbes to regulate the liver physiology through the intestinal liver axis (11).

Epigallocatechin gallate (EGCG) is one of the most abundant catechins in green tea and is a typical polyphenol flavonoid compound that has eight free hydroxyl groups (12). Extensive studies have shown that EGCG is the most widely active substance in green tea, with antibacterial, antiviral, anti-oxidant, anti-arteriosclerosis, anti-thrombosis, anti-vascular proliferation, anti-inflammatory, and anti-tumor effects (12, 13). Cancer is the result of the final pathogenic development of inflammation (14). Many inflammatory factors, including pro-inflammatory factors and chemokines, mediate the occurrence of cancer (15, 16). In inhibiting inflammation and cancer, EGCG has shown good performance significantly inhibiting inflammatory cell expression in various cancer cells (17–19). Clinical studies have shown that EGCG can resist the proliferation and migration of human cancer cells by inhibiting the expression of *NF-κB* and *MMP-9* (20) On the other hand, EGCG can maintain the integrity of the epithelial barrier of Caco-2 cells and inhibit the increase in the intestinal epithelial permeability induced by *IFN-γ* (17). After the gut microbes (including *Enterobacter aerogenes*, *Raoultella planticola*, *Klebsiella pneumoniae*) hydrolysis EGCG, the host gut can absorb these microbial metabolites (21). According to previous studies, after EGCG is hydrolyzed in the intestine, a series of transformations and degradations will occur. Studies have shown that 5-(3,5-dihydroxyphenyl)-4-hydroxyvaleric acid is the main metabolite of EGCG in the intestine, and, after the intestinal microbes degrade EGCG, 5-(3’,5’-dihydroxyphenyl) -γ-valerolactone is absorbed by the body, and its glucuronic acid form is the main urinary metabolite (22). EGCG is first absorbed by the intestine after being ingested, and, in this process, the intestinal flora plays a vital role (23). On the other hand, EGCG also has a significant regulatory effect on the composition of the intestinal microbial community. The addition of EGCG to the diet can reduce the microbial abundances of *Alipipes*, *Anaerotruncu*, and *Desulfovibrio*, which are related to metabolic disorders, and can remedy the malnutrition of the intestinal flora caused by non-alcoholic steatohepatitis. EGCG can also inhibit *Prevo* and *Fusobacterium* to regulate the intestinal flora (17). Therefore, we assume that EGCG can maintain the gut microbial community’s structural homeostasis and serum metabolites to reduce an acute host injury. This study explored the protection of EGCG in mice with an acute injury induced by lipopolysaccharide, observed the changes in the intestinal flora and serum metabolites of these mice, and determined the protective effect of EGCG.

## Materials and Methods

### Animals and Experimental Design

**Figure 1:**
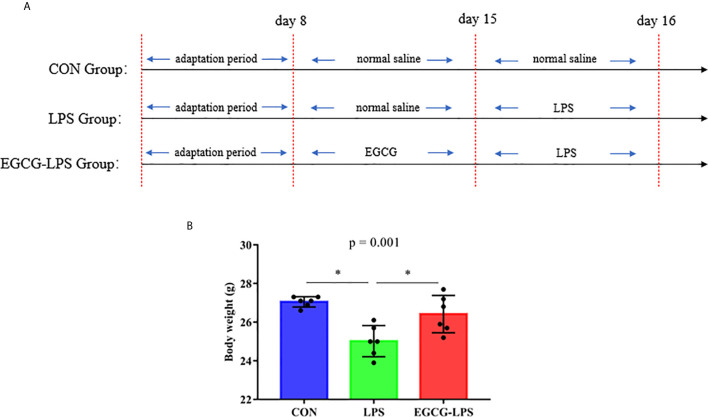
The protective effects of intragastric administration of EGCG on body weight. **(A)** the experimental process; **(B)** body weight. Data are mean ± SD (n = 6) and analyzed by one-way ANOVA. “*” means that the horizontal line connects the *p*-value of the data between the two groups <0.05, and the “*P*” value in the figure represents the accurate value of *p* between the three groups of data, and some of the p values between the three groups are too small to be expressed as *p*<0.0001.

Animal experiments were performed according to the Guidelines for the Care and Use of Laboratory Animals of Hunan Agricultural University. We selected 18 eight-week-old ICR mice (Hunan Sileike Jingda Co, Changsha, China) for a week of adaptation. On the eighth day, we randomly divided them into three groups: the control group (CON), the LPS model (LPS), and the EGCG gavage treatment group (EGCG-LPS). On days 8–15 of the experiment, according to the pre-experiment results (**Supplemental Figures 1, 2**), we gave the EGCG-LPS group a gavage of EGCG (200 mg/kg), and the other two groups received normal saline gavage of the same dose. On the 15th day of the experiment, we administered LPS (15 mg/kg) intraperitoneal injections in the mice in the LPS and EGCG-LPS groups as well as intraperitoneal injections of normal saline of the same dose in the CON group (**Figure 1A**). On the 16th day, all mice were sacrificed, serum was collected for metabolome analysis, a portion of the liver and jejunum were fixed in 4% formalin for histomorphological analysis, and a portion of the liver and colon contents were taken, quick-frozen in liquid nitrogen, and used for liver index determination and 16srDNA determination.

### Jejunum and Liver Histopathology

The tissue fixed in 4% formalin was treated with gradient concentrations of xylene and ethanol and then embedded in paraffin. The embedded sample was dehydrated and transparent; it was dyed with hematoxylin and eosin and then covered with neutral gum. The specific process was based on our previous research (24).

### Determination of Proinflammatory Factors and Oxidative Stress Substances in Liver Tissue

The concentrations of *TNF-α, IL-1β, IL-6, MCP-1, MIP-2, IFN-γ* and MPO in the liver tissue samples were measured by a Mouse ELISA Kit (Jiangsu Yutong Biological Technology Co) with a microplate reader (Bio Tek, USA). The concentration of NO as well as the enzyme activity of ALT and AST in the liver tissue samples were respectively measured by a Nitric Oxide (NO) assay kit (Microwell plate method), Alanine aminotransferase Assay Kit and Aspartate aminotransferase Assay Kit (Nanjing Jiancheng Bioengineering Institute) with a microplate reader (Bio Tek, USA).

### Serum Metabolomic Analyses

We extracted the serum with methanol and add dichlorophenylalanine. After mixing the liquid, we centrifuged it to obtain the supernatant and perform an on-machine test of LC-MS. The specific chromatographic column and liquid phase conditions used referred to our previous research. The off-machine data was preprocessed with Compound Discoverer software to obtain the molecular weight, mass-to-charge ratio, retention time, and peak area. Finally, we used SIMCA14.1 to perform orthogonal partial least squares discrimination analysis on the data to obtain the VIP value. We used the Human Metabolome Database (https://hmdb.ca/spectra/ms/search) for substance comparison.

### 16S Ribosomal RNA Amplicon Sequencing

We used the QIAamp DNA Stool Mini Kit to extract microbial genomic DNA from the colon contents. We selected the V3-V4 region sequence of 16S rDNA for high-throughput sequencing analysis on the Illumina platform. The specific process was based on our previous research (24). We then used Trimmomatic software to perform quality control and optimization of the off-machine data before finally performing operational taxonomic unit (OTU) clustering to determine the composition of the microbial communities at different taxonomic levels. Additionally, we used mothur (Version 1.33.3) to perform alpha diversity analysis. The composition of the microbial community was generated by BMKCloud (http://www.biocloud.net/). The abundance of the sequenced microorganisms at different taxonomic levels was uploaded to http://huttenhower.sph.harvard.edu/galaxy for linear discriminant analysis (LDA) and LDA effect size (LEfSe) analysis.

### Data Analysis

All of the data in this study are expressed as mean ± standard deviation (SD). The data were analyzed using SPSS 22.0. The differences between the means of the experimental groups were analyzed using one-way analysis of variance and Tukey’s multiple comparison test. A *p* value < 0.05 was regarded as a significant difference.

## Results

### EGCG Improves the Acute Injury Induced by LPS in Mice

Before the last intraperitoneal injection of LPS, we administered EGCG gavage for 7 consecutive days. The results showed that: compared with the CON group, the intraperitoneal injection of LPS significantly reduced the weight of the mice. But the mice that received EGCG gavage treatment, compared with the LPS group, the weight was significantly increased and avoid the weight loss of mice (**Figure 1B**, *p <* 0.05).

### EGCG Inhibits the Development of Acute Injury Induced by LPS

**Figure 2:**
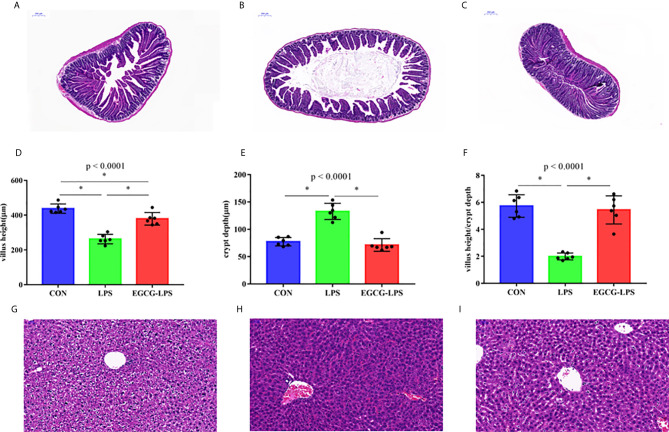
The protective effect of intragastric administration of EGCG on jejunum tissue and liver tissue damage. Images of colon morphology in the CON **(A)**, LPS **(B)**, and LPS-EGCG **(C)** groups under 200× visual fields; villus height **(D)**, crypt depth **(E)** and villus height/crypt depth **(F)** in the three groups; Images of liver morphology in the CON **(G)**, LPS **(H)**, and EGCG-LPS **(I)** groups under 200× visual fields; Data are mean ± SD (n = 6) and analyzed by one-way ANOVA. “*” means that the horizontal line connects the *p*-value of the data between the two groups <0.05, and the “*P*” value in the figure represents the accurate value of *p* between the three groups of data, and some of the p values between the three groups are too small to be expressed as *p*<0.0001.

The results of jejunum histomorphology analysis (**Figures 2A–F**) showed that, compared with the CON group, the intraperitoneal injection of LPS caused a significant decrease in the height of the mouse jejunum villi and a significant increase in the depth of the crypts (*p* < 0.05). However, EGCG-LPS group, compared with the LPS group, the mice had significantly increased villus heights, while the crypt depth was reduced considerably (*p* < 0.05). It returned to a normal level similar to that of the CON group. The morphological results of the liver tissue (**Figures 2G–I**) showed that the liver cells in the CON group were arranged regularly and neatly centered around the central vein. The liver cells in the LPS group showed apparent inflammatory damage, and the cells were arranged irregularly and exhibited inflammatory infiltration. These damages phenomena were greatly alleviated in the EGCG-LPS group.

### EGCG Inhibits Inflammation and Oxidative Stress in Liver Tissue

**Figure 3:**
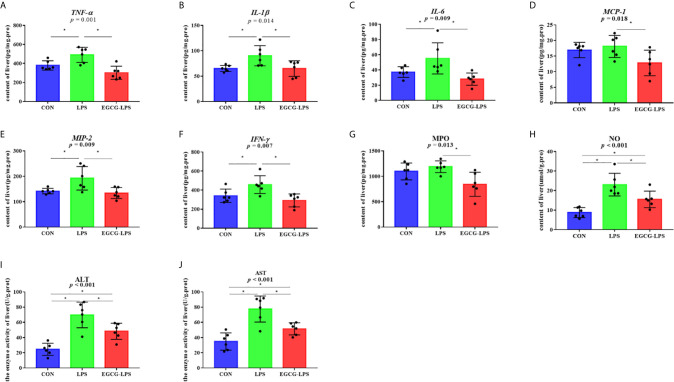
The inhibition of the intragastric administration of EGCG on inflammation and oxidative stress in liver tissue. **(A)** the concentration of *TNF-α* in liver tissue; **(B)** the concentration of *IL-1β* in liver tissue; **(C)** the concentration of *IL-6* in liver tissue; **(D)** the concentration of *MCP-1* in liver tissue; **(E)** the concentration of *MIP-2* in liver tissue; **(F)** the concentration of *IFN-γ* in liver tissue; **(G)** the concentration of MPO in liver tissue; **(H)** the concentration of total NO in liver tissue; **(I)** the enzyme activity of ALT in liver tissue; **(J)** the enzyme activity of AST in liver tissue. Data are mean ± SD (n = 6) and analyzed by one-way ANOVA.”*” means that the horizontal line connects the *p*-value of the data between the two groups <0.05, and the “*P*” value in the figure represents the accurate value of *p* between the three groups of data, and some of the p values between the three groups are too small to be expressed as *p*<0.0001.

We measured the concentration of inflammatory factors in the liver tissues (**Figures 3A–F**), and the results showed that, when compared with the CON group, the intraperitoneal injection of LPS significantly increased the concentration of *TNF-α, IL-1β, IL-6, MIP-2*, and *IFN-γ* in the liver tissue (p < 0.05). However, in the EGCG-LPS group received EGCG gavage protection, compared with the LPS group, the concentrations of *TNF-α, IL-1β, IL-6, MCP-1, MIP-2, IFN-γ* in the liver tissues of the mice were significantly reduced (p < 0.05). The oxidative stress index of the mouse liver tissue (**Figures 3G–J**) showed that, when compared with the CON group, the concentration of NO as well as the enzyme activity of ALT and AST in the liver tissue of the LPS group increased significantly (p < 0.05). Compared with the LPS group, the concentration of NO and MPO as well as the enzyme activity of ALT and AST in the mice’s liver tissue after receiving the EGCG treatment decreased significantly (p < 0.05). However, the alleviation of this oxidative stress index did not return to the same level as the CON group.

### EGCG Regulates Intestinal Microbes in Mice With Acute Injury Induced by LPS

**Figure 4:**
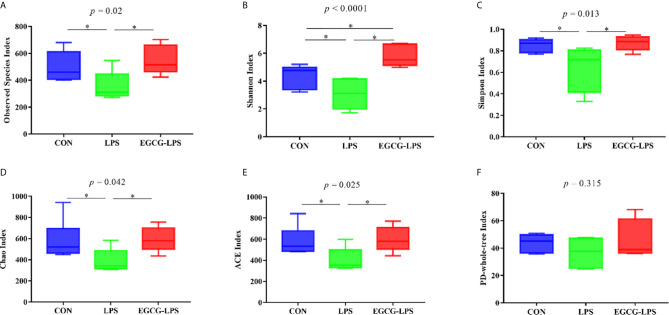
The protective effect of the intragastric administration of EGCG on mouse gut microbial diversity. **(A)** Observed Species; **(B)** Shannon index; **(C)** Simpson index; **(D)** Chao index; **(E)** ACE index; **(F)** PD-whole-tree index. Data are mean ± SD (n = 6) and analyzed by one-way ANOVA. “*” means that the horizontal line connects the *p*-value of the data between the two groups <0.05, and the “*P*” value in the figure represents the accurate value of *p* between the three groups of data, and some of the p values between the three groups are too small to be expressed as *p*<0.0001.

We first analyzed the 16S rRNA *V3-V4* region sequence of colonic microorganisms to obtain the α diversity of intestinal microorganisms (**Figures 4A–F**). Compared with the CON group, the observed species, Shannon index, Simpson index, Chao index, and ACE index in the LPS group all decreased significantly (*p* < 0.05). However, in the mice that received the EGCG treatment, compared with the LPS group, the observed species, Shannon index, Simpson index, Chao index, and ACE index were significantly improved (*p* < 0.05). Therefore, EGCG gavage can significantly protect mice from the decrease in intestinal microbial diversity caused by LPS.

**Figure 5:**
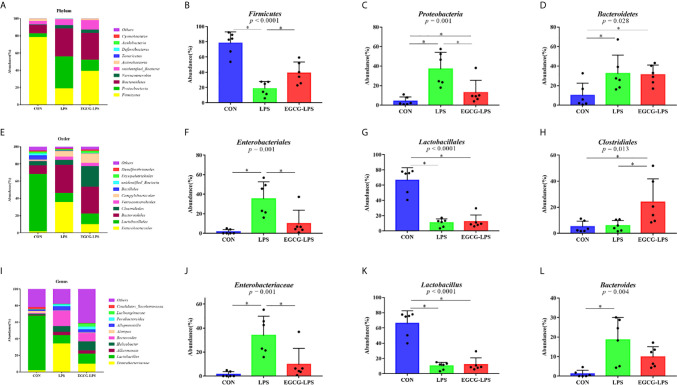
The protective effect of the intragastric administration of EGCG on the intestinal microflora of mice. **(A)** Relative abundance of gut microbiota phyla; **(B)** percentage of *Firmicutes* in each sample from the three groups; **(C)** percentage of *Proteobacteria* in each sample from the three groups; **(D)** percentage of *Bacteroidetes* in each sample from the three groups; **(E)** relative abundance of gut microbiota orders; **(F)** percentage of *Enterobacteriales* in each sample from the three groups; **(G)** percentage of *Lactobacillales* in each sample from the three groups; **(H)** percentage of *Clostridiales* in each sample from the three groups; **(I)** relative abundance of gut microbiota genus; **(J)** percentage of *Enterobacteriaceae* in each sample from the three groups; **(K)** percentage of *Lactobacillus* in each sample from the three groups; **(L)** percentage of *Bacteroides* in each sample from the three groups. *p<0.05”*” means that the horizontal line connects the *p*-value of the data between the two groups <0.05, and the “*P*” value in the figure represents the accurate value of *p* between the three groups of data, and some of the p values between the three groups are too small to be expressed as *p*<0.0001.

The microbial community composition of the mouse colon (**Figures 5A–D**) showed that *Firmicutes*, *Proteobacteria*, and *Bacteroidetes* were the three main microorganisms at the phylum level. The abundance of these three microorganisms accounts for more than 85% of all microorganisms. The *Firmicutes* abundance in the CON, LPS, and EGCG-LPS groups were 78.24656%, 18.58981%, and 39.05929%, respectively. *Proteobacteria* abundance accounted for 4.079801%, 37.06216%, and 12.85193%, respectively. The *Bacteroidetes* abundance was 9.898671%, 32.38386%, and 31.01083%, respectively. Compared with the CON group, the abundance of *Firmicutes* in the LPS group was significantly reduced (p < 0.05), but the abundance of *Proteobacteria* and *Bacteroidetes* was significantly increased (p < 0.05). Compared with the LPS group, the abundance of *Firmicutes* in the EGCG-LPS group was significantly increased (p < 0.05), but the abundance of *Proteobacteria* was significantly reduced (p < 0.05).

The microbial community composition of the mouse colon (**Figures 5E–H**) showed that *Lactobacillales*, *Bacteroidales*, and *Clostridiales* were the three main microorganisms at the order level. The abundance of these three microorganisms accounts for more than 80% of all microorganisms. The *Lactobacillales* abundance in the CON, LPS, and EGCG-LPS groups were 66.42404%, 10.58623%, and 12.08513%, respectively. The *Bacteroidales* abundance was 9.873076%, 32.36142%, and 30.93685%, respectively. The *Clostridiales* abundance was 5.006486%, 5.806248%, and 24.00442%, respectively. Compared with the CON group, the abundance of *Lactobacillales* in the LPS group was significantly reduced (p < 0.05), but the abundance of *Enterobacteriales* was significantly increased (p < 0.05). Compared with the LPS group, the abundance of *Clostridiales* in the EGCG-LPS group was significantly increased (p < 0.05), but the abundance of *Enterobacteriales* was significantly reduced (p < 0.05).

The microbial community composition of the mouse colon (**Figures 5I–L**) showed that *Enterobacteriaceae*, *Lactobacillus*, *Akkermansia*, *Helicobacter*, and *Bacteroides* were the five main microorganisms at the genus level. The abundance of these five microorganisms accounts for more than 70% of all microorganisms. The *Enterobacteriaceae* abundance in the CON, LPS, and EGCG-LPS groups was 1.453315%, 33.92974%, and 9.697065%, respectively. The *Lactobacillus* abundance was 66.11865%, 10.09782%, and 11.92314%, respectively. The *Akkermansia* abundance was 0.545563%, 3.846639%, and 3.939904%, respectively. The *Helicobacter* abundance was 1.53536%, 6.888258%, and 10.97262%, respectively. The *Bacteroides* abundance was 1.146524%, 18.53617%, and 9.785772%, respectively. Compared with the CON group, the abundance of *Lactobacillus* in the LPS group was significantly reduced (p < 0.05), but the abundances of *Enterobacteriaceae* and *Bacteroides* were significantly increased (p < 0.05). Compared with the LPS group, the abundance of *Enterobacteriaceae* in the EGCG-LPS group was significantly reduced (p < 0.05).

**Figure 6:**
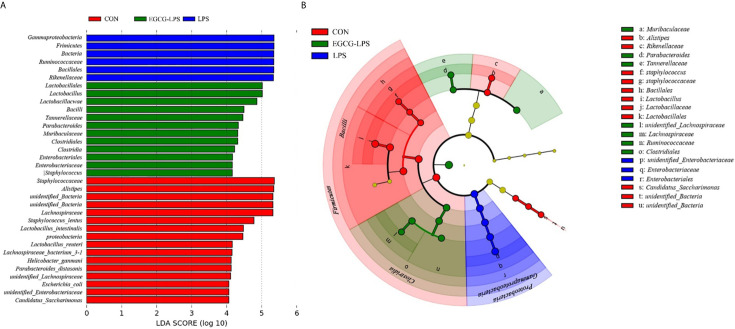
Characteristic of mouse colon microorganisms determined by intragastric administration of EGCG. **(A)** LDA score, an LDA score higher than 4 was considered to be an important contributor to the model; **(B)** LEfSe taxonomic cladogram, different colors suggest enrichment of certain taxa in the control group (red), LPS group (blue) and LPS-EGCG group (green).

We used linear discriminant analysis (LDA) to estimate the effect of the abundance of each component (species) on the difference effects (**Figure 6A**). LEfSe analysis (**Figure 6B**) showed that the CON group was significantly enriched in *Lactobacillus*, *Lactobacillaceae* and *Lactobacillales*. The LPS group was significantly enriched in *Enterobacteriaceae* and *Enterobacteriales*. Meanwhile, the EGCG-LPS group was significantly enriched in *Clostridiales*.

### EGCG Affects the Serum Metabolomic Profiles During Acute Injury

**Figure 7:**
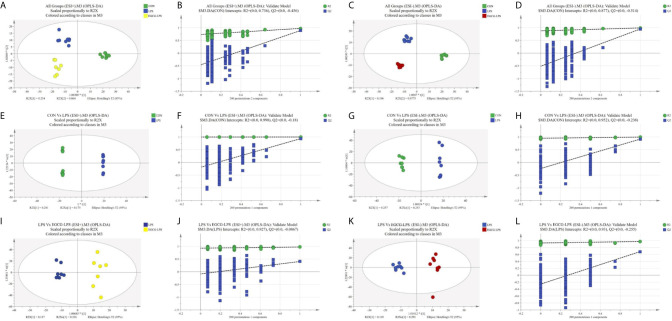
Plots of the multivariate statistical comparisons between groups. **(A)** OPLS-DA score plot of all groups (ESI+); **(B)** OPLS-DA (Validate Model) score plot of all groups (ESI+); **(C)** OPLS-DA score plot of all groups (ESI-); **(D)** OPLS-DA (Validate Model) score plot of all groups (ESI-); **(E)** OPLS-DA score plot of CON Vs LPS (ESI+); **(F)** OPLS-DA (Validate Model) score plot of CON Vs LPS (ESI+); **(G)** OPLS-DA score plot of CON Vs LPS (ESI-); **(H)** OPLS-DA (Validate Model) score plot of CON Vs LPS (ESI-); **(I)** OPLS-DA score plot of LPS Vs LPS-EGCG (ESI+); **(J)** OPLS-DA (Validate Model) score plot of LPS Vs LPS-EGCG (ESI+); **(K)** OPLS-DA score plot of LPS Vs LPS-EGCG (ESI-); **(L)** OPLS-DA (Validate Model) score plot of LPS Vs LPS-EGCG (ESI-).

**Figure 8:**
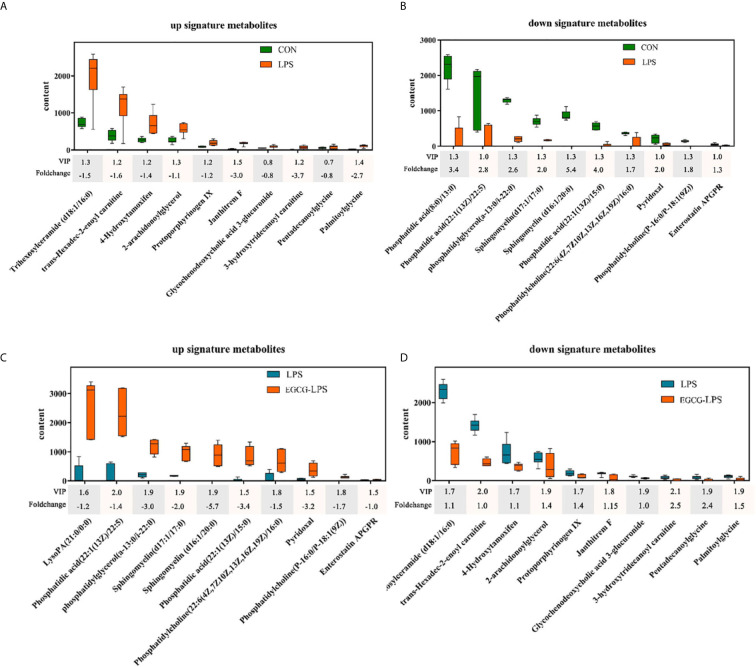
The regulatory effect of the intragastric administration of EGCG on mouse serum metabolites. **(A)** Compared with the CON group, ten characteristic metabolites increased in the LPS group; **(B)** Compared with the CON group, ten characteristic metabolites reduced in the LPS group; **(C)** Compared with the LPS group, ten characteristic metabolites increased in the EGCG-LPS group; **(D)** Compared with the LPS group, ten characteristic metabolites reduced in the EGCG-LPS group.

We used GC-MS to determine the serum metabolites from the acute injury induced by LPS in the mice. First, we used orthogonal partial least squares discrimination analysis (OPLS-DA) to establish a model of the relationship between metabolite expression and sample category to predict the sample category. At the same time, we also verified this model. The results showed that the serum metabolites of the different groups changed significantly, and the similarity of these metabolisms within each group was high (**Figure 7**). We specifically analyzed the different metabolites between the CON and LPS groups. We found that the serum levels of the Trihexosylceramide (d18:1/16:0), trans - Hexadec - 2 - enoyl carnitine, 4 - Hydroxytamoxifen, 2 - arachidonoylglycerol, Protoporphyrinogen IX, Janthitrem F, Glycochenodeoxycholic acid 3 - glucuronide, 3 - hydroxytridecanoyl carnitine, Pentadecanoylglycine and Palmitoylglycine significantly increased after the acute injury induced *via* LPS in the mice (**Figure 8A**, p < 0.05). On the other hand, the injection of LPS significantly reduced the phosphatidic acid (18:0/13:0), phosphatidic acid (22):1(13Z)/22:5), phosphatidylglycerol (a-13:0/i-22:0), sphingomyelin (d17:1/17:0), sphingomyelin (d16:1/20:0), phosphatidic acid (22:1(13Z)/15:0), phosphatidylcholine (22:6(4Z,7Z10Z,13Z,16Z,19Z)/16:0), pyridoxal, phosphatidylcholine (P-16:0/P-18:1(9Z)) as well as the enterostatin APGPR content (**Figure 8B**, p < 0.05). However, under the protection of EGCG gavage, the serum metabolites of mice were not greatly affected by the acute stress induced by LPS (**Figures 8C**, **D**, p < 0.05).

## Discussion

This study explored the protective effect of EGCG on LPS-induced acute injury. We observed that EGCG can significantly prevent the morphological damage done to the jejunum and liver caused by LPS. At the same time, EGCG gavage treatment can also significantly inhibit LPS-induced liver inflammation and oxidative stress. The 16srDNA results show that EGCG can maintain the structure of the intestinal microbial community and inhibit the abundance of enteric pathogens, such as *Enterobacteriaceae*. Via serum metabolomics analysis, we posit that glycerophospholipid and sphingolipid substances are promoted by EGCG, thereby improving the organism’s immune function.

Polyphenols have been widely used to improve the We found that the gavage of EGCG significantly increased the content of pyridoxal in the serum of the mice, thereby enhancing their ability to reduce the expression of *IL-1β, TNF-α, IL-6*, and other pro-inflammatory factors while also enhancing the organisms’ immunity due to their good biological activity (25–27). Studies have shown that polyphenols, including EGCG and theaflavin-3′-O-gallate (TF3′G), are absorbed in the jejunum after being ingested (28). Therefore, the nutrient absorption capacity of the jejunum is particularly important for the bioavailability of EGCG. T The jejunal epithelium can be renewed in three days, and the cells on the intestinal villi are replaced *via* the proliferation of crypt cells (29). The role of the intestinal villi is to increase the area of absorption while also promoting absorption (30). Extensive studies have shown that the acute damage done by LPS to the organism can lead to the destruction of the intestinal integrity and barrier function (31, 32). Our research shows that gavage treatment with EGCG can protect the jejunum’s structural integrity and its ability to absorb nutrients so that it will not be affected by an acute injury caused by LPS. When LPS enters the organism, *TLR4* in the liver cells can actively recognize LPS and activate the expression of downstream pro-inflammatory factors (33). Pro-inflammatory factors, including the *IFN-γ, TNF-α, IL-17, and IL-1* family are widely expressed in the liver, which further deepens the inflammatory damage done to the liver (34). Our research shows that EGCG can well inhibit LPS from activating *TNF-α, IL-1β, IL-6, MCP-1, MIP-2*, and *IFN-γ*. Although the concentration of *MCP-1* and MPO was not statistically significant between the CON and LPS groups, from a data point of view, these two indicators still showed an upward trend between these two groups, which proves that LPS induced the occurrence of acute stress. Moreover, under the protection of EGCG, these two indicators were significantly reduced when compared with the LPS group, and, at the same time, there was no significant difference between the EGCG group and CON group.

After being injured, the liver will feedback this stress to the intestine through the portal vein, and this process reshapes the structure of the intestinal microbial community (35). Henao-Mejia’s research team found that the immersion of a large number of Toll-like receptors in the portal vein can significantly promote the expression of *TNF-α* and further induce the development of liver inflammation damage (36). After this signal pathway is transmitted from the portal vein into the intestine, it leads to the intestinal microbes’ structural changes. *Bacterial* genera, *bacteroides*, and *Ruminococcus* are significantly increased, while *Prevotella* is significantly reduced (37). Our research also found that the intraperitoneal injection of LPS significantly increased the abundance of *bacterial genera* and *Bacteroides*. Although the gavage of EGCG failed to inhibit the increase in the abundance of *Bacteroides*, it did regulate *Firmicutes*/*Bacteroidetes*. This ratio is also a characteristic of the stability of the intestinal microbial community (38). However, EGCG treatment given by oral gavage did not prevent the decrease in the abundance of *Lactobacillales*, similarly to the previous report showing that diet supplementation with EGCG has a small effect on the abundance of *Bifidobacteria* (39). Gut microbes play an irreplaceable role in intestinal immunity. *Proteobacteria* is the intestinal microorganism known to be the most relevant to diseases (40). Most of the microorganisms belonging to this phylum level are human pathogens. An increasing number of studies have found that *Proteobacteria* is an important cause of intestinal inflammation and metabolic disease (41, 42). In this experiment, the gavage treatment of EGCG significantly reduced the abundance of *Proteobacteria* underwater at the portal level, thereby reducing the risk of intestinal inflammation in the mice. *Enterobacteriales* is an order under *Gamma-proteobacteria*, which is under *Proteobacteria.* The gavage treatment of EGCG also suppressed the abundance of such pathogenic bacteria. EGCG can inhibit the expression of *stx1* and *RecA* in enterobacteria membrane cells and induce the expression of *stx2* and *oxyR* by increasing the oxidative stress response, thereby increasing the permeability of the outer membrane and leading to the inactivation of *Enterobacter* (43).

As an important part of systems biology, metabonomics is used in the prediction of the effects of diseases and tumor drugs as well as the detection of markers (44). Sphingolipids are an essential part of the structure of biological membranes, and an increasing number of studies have shown that they are closely related to immunity and inhibiting inflammation (45, 46). Sphingomyelin metabolites can reduce the ability of TNF to induce CCL5 and minimize the risk of inflammation. Our research showed that, under the protection of EGCG gavage, the serum levels of sphingomyelin (d17:1/17:0) and sphingomyelin (d16:1/20:0) were not affected by the LPS-induced acute injury. Pyridoxal is an active form of vitamin B6, which has always been regarded as an important substance for improving immune function and reducing inflammation (47). We found that the gavage of EGCG significantly increased the content of pyridoxal in the serum of the mice, thereby enhancing their ability to reduce the expression of *IL-1β, TNF-α, IL-6*, and other pro-inflammatory factors while also enhancing the organisms’ immunity. More importantly, previous studies have shown that vitamins have an inhibitory effect on the *Stx2* production of *Enterobacteriales*, which reduces the growth and virulence potential of this type of intestinal pathogenic bacteria (48). This corresponds to the fact that under the protection of EGCG gavage in our experiment, the abundance of *Enterobacteriales* in the colon is significantly reduced. On the other hand, the reduction of harmful substances also reflects the effect of EGCG gavage therapy, which significantly reduced the content of trans-Hexadec-2-enoyl carnitine in the serum of the mice with LPS-induced inflammation. Trans-Hexadec-2-enoyl carnitine is a kind of acylcarnitine. The accumulation of acylcarnitine in the body promotes the expression of pro-inflammatory factors in the organism and induces the phosphorylation of JNK and ERK (49). Studies have also found that an increased concentration of acylcarnitine increases the risk of intestinal permeability. It can be seen that the gavage treatment of EGCG significantly increased the content of immune-enhancing substances in the blood and inhibited the connection between harmful substances and inflammation.

## Conclusions

Our experimental results show that the gavage of EGCG can effectively prevent the negative effects of LPS-induced acute injury. On the other hand, EGCG can stabilize the intestinal microbial community structure of mice in general and reduce pathogenic bacteria, such as *Proteus* and *Enterobacter*. Moreover, EGCG can regulate serum metabolite components; promote the production of beneficial metabolomes, including sphingomyelin (d17:1/17:0), sphingomyelin (d16:1/20:0); and reduce the production of these harmful metabolomes, including trans-Hexadec-2-enoyl carnitine. Therefore, EGCG can significantly inhibit the acute injury induced by LPS and improve the immune function of the organism.

## Data Availability Statement

The datasets presented in this study can be found in online repositories. The names of the repository/repositories and accession number(s) can be found below: https://www.ncbi.nlm.nih.gov/, SUB8703003.

## Ethics Statement

The animal study was reviewed and approved by Animal Care and Use Committee of Hunan Agricultural University.

## Author Contributions

YM performed the study and conducted data analysis. GL designed the research. MT provided assistance for the study. HJ and JF prepared the first draft of the manuscript. All authors contributed to the article and approved the submitted version.

## Funding

This research was supported by National Natural Science Foundation of China (No. 31772642, 31672457, 41807135), Local Science and Technology Development Project Guided by The Central Government (YDZX20184300002303, 2018CT5002), and Hunan Provincial Science and Technology Department (2019TP2004, 2018WK4025, 2020NK2004, 2020ZL2004, 2016NK2101, 2016TP2005, 2018CT5002), China Postdoctoral Science Foundation (2018M632963, 2019T120705), Scientific Research Fund of Hunan Provincial Education Department (2020JGYB112, 18B107), Double first-class construction project of Hunan Agricultural University (SYL201802003, YB2018007, CX20190497), and Natural Science Foundation of Hunan province, China (No. 2019JJ50220).

## Conflict of Interest

The authors declare that the research was conducted in the absence of any commercial or financial relationships that could be construed as a potential conflict of interest.

## References

[1] Cribbs SK Martin GS. Expanding the global epidemiology of sepsis. Crit Care Med (2007) 35(11):2646–8. 10.1097/01.Ccm.0000288082.99980.90 18075373

[2] Geng C Guo Y Wang C Cui C Han W Liao D. Comprehensive Evaluation of Lipopolysaccharide-Induced Changes in Rats Based on Metabolomics. J Inflammation Res (2020) 13:477–86. 10.2147/jir.S266012 PMC7457572 32904659

[3] Romerio A Peri F. Increasing the Chemical Variety of Small-Molecule-Based TLR4 Modulators: An Overview. Front Immunol (2020) 11:1210. 10.3389/fimmu.2020.01210 32765484 PMC7381287

[4] Vanaja SK Russo AJ Behl B Banerjee I Yankova M Deshmukh SD. Bacterial Outer Membrane Vesicles Mediate Cytosolic Localization of LPS and Caspase-11 Activation. Cell (2016) 165(5):1106–19. 10.1016/j.cell.2016.04.015 PMC4874922 27156449

[5] Huber RG Berglund NA Kargas V Marzinek JK Holdbrook DA Khalid S. A Thermodynamic Funnel Drives Bacterial Lipopolysaccharide Transfer in the TLR4 Pathway. Structure (London Engl 1993) (2018) 26(8):1151–61.e4. 10.1016/j.str.2018.04.007 29779787

[6] Strnad P Tacke F Koch A Trautwein C. Liver - guardian, modifier and target of sepsis. Nat Rev Gastroenterol Hepatol (2017) 14(1):55–66. 10.1038/nrgastro.2016.168 27924081

[7] Mandrekar P Szabo G. Signalling pathways in alcohol-induced liver inflammation. J Hepatol (2009) 50(6):1258–66. 10.1016/j.jhep.2009.03.007 PMC3342816 19398236

[8] Hu C Zhao L Shen M Wu Z Li L. Autophagy regulation is an effective strategy to improve the prognosis of chemically induced acute liver injury based on experimental studies. J Cell Mol Med (2020) 24(15):8315–25. 10.1111/jcmm.15565 PMC7412417 32627386

[9] Yu LX Schwabe RF. The gut microbiome and liver cancer: mechanisms and clinical translation. Nat Rev Gastroenterol Hepatol (2017) 14(9):527–39. 10.1038/nrgastro.2017.72 PMC6467288 28676707

[10] De Minicis S Rychlicki C Agostinelli L Saccomanno S Candelaresi C Trozzi L. Dysbiosis contributes to fibrogenesis in the course of chronic liver injury in mice. Hepatol (Baltimore Md) (2014) 59(5):1738–49. 10.1002/hep.26695 23959503

[11] Acharya C Bajaj JS. Altered Microbiome in Patients With Cirrhosis and Complications. Clin Gastroenterol Hepatol Off Clin Pract J Am Gastroenterol Assoc (2019) 17(2):307–21. 10.1016/j.cgh.2018.08.008 PMC6314917 30099098

[12] Chakrawarti L Agrawal R Dang S Gupta S Gabrani R. Therapeutic effects of EGCG: a patent review. Expert Opin Ther Patents (2016) 26(8):907–16. 10.1080/13543776.2016.1203419 27338088

[13] Liu B Yan W. Lipophilization of EGCG and effects on antioxidant activities. Food Chem (2019) 272:663–9. 10.1016/j.foodchem.2018.08.086 30309596

[14] Mantovani A Allavena P Sica A Balkwill F. Cancer-related inflammation. Nature (2008) 454(7203):436–44. 10.1038/nature07205 18650914

[15] Coussens LM Werb Z. Inflammation and cancer. Nature (2002) 420(6917):860–7. 10.1038/nature01322 PMC2803035 12490959

[16] Diakos CI Charles KA McMillan DC Clarke SJ. Cancer-related inflammation and treatment effectiveness. Lancet Oncol (2014) 15(11):e493–503. 10.1016/s1470-2045(14)70263-3 25281468

[17] Gil-Cardoso K Ginés I Pinent M Ardévol A Blay M Terra X. Effects of flavonoids on intestinal inflammation, barrier integrity and changes in gut microbiota during diet-induced obesity. Nutr Res Rev (2016) 29(2):234–48. 10.1017/s0954422416000159 27841104

[18] Jhang JJ Lu CC Yen GC. Epigallocatechin gallate inhibits urate crystals-induced peritoneal inflammation in C57BL/6 mice. Mol Nutr Food Res (2016) 60(10):2297–303. 10.1002/mnfr.201600106 27234527

[19] Rajagopal C Lankadasari MB Aranjani JM Harikumar KB. Targeting oncogenic transcription factors by polyphenols: A novel approach for cancer therapy. Pharmacol Res (2018) 130:273–91. 10.1016/j.phrs.2017.12.034 29305909

[20] Luo KW Wei C Lung WY Wei XY Cheng BH Cai ZM. EGCG inhibited bladder cancer SW780 cell proliferation and migration both in vitro and in vivo via down-regulation of NF-κB and MMP-9. J Nutr Biochem (2017) 41:56–64. 10.1016/j.jnutbio.2016.12.004 28040581

[21] Gan RY Li HB Sui ZQ Corke H. Absorption, metabolism, anti-cancer effect and molecular targets of epigallocatechin gallate (EGCG): An updated review. Crit Rev Food Sci Nutr (2018) 58(6):924–41. 10.1080/10408398.2016.1231168 27645804

[22] Liu Z de Bruijn WJC Bruins ME Vincken JP. Microbial Metabolism of Theaflavin-3,3’-digallate and Its Gut Microbiota Composition Modulatory Effects. J Agric Food Chem (2021) 69(1):232–45. 10.1021/acs.jafc.0c06622 PMC7809692 33347309

[23] Liu AB Tao S Lee MJ Hu Q Meng X Lin Y. Effects of gut microbiota and time of treatment on tissue levels of green tea polyphenols in mice. BioFactors (Oxford England) (2018) 44(4):348–60. 10.1002/biof.1430 PMC6222019 29740891

[24] Ma Y Hu C Yan W Jiang H Liu G. Lactobacillus pentosus Increases the Abundance of Akkermansia and Affects the Serum Metabolome to Alleviate DSS-Induced Colitis in a Murine Model. Front Cell Dev Biol (2020) 8:591408. 10.3389/fcell.2020.591408 33195257 PMC7609924

[25] Ding S Jiang H Fang J. Regulation of Immune Function by Polyphenols. J Immunol Res (2018) 2018:1264074. 10.1155/2018/1264074 29850614 PMC5925142

[26] Wang K Jin X Li Q Sawaya A Le Leu RK Conlon MA. Propolis from Different Geographic Origins Decreases Intestinal Inflammation and Bacteroides spp. Populations in a Model of DSS-Induced Colitis. Mol Nutr Food Res (2018) 62(17):e1800080. 10.1002/mnfr.201800080 29889351

[27] Ding S Xu S Fang J Jiang H. The Protective Effect of Polyphenols for Colorectal Cancer. Front Immunol (2020) 11:1407. 10.3389/fimmu.2020.01407 32754151 PMC7366338

[28] Nguyen HN Tanaka M Li B Ueno T Matsuda H Matsui T. Novel in situ visualisation of rat intestinal absorption of polyphenols via matrix-assisted laser desorption/ionisation mass spectrometry imaging. Sci Rep (2019) 9(1):3166. 10.1038/s41598-019-39405-w 30816166 PMC6395804

[29] Jeurissen SH Lewis F van der Klis JD Mroz Z Rebel JM ter Huurne AA. Parameters and techniques to determine intestinal health of poultry as constituted by immunity, integrity, and functionality. Curr Issues Intestinal Microbiol (2002) 3(1):1–14. 12022808

[30] Zheng Z Zuo Z Zhu P Wang F Yin H Peng X. A study on the expression of apoptotic molecules related to death receptor and endoplasmic reticulum pathways in the jejunum of AFB(1)-intoxicated chickens. Oncotarget (2017) 8(52):89655–64. 10.18632/oncotarget.20333 PMC5685699 29163778

[31] Chang Y Deng Q Zhang Z Zhao H Tang J Chen X. Glucagon-like peptide 2 attenuates intestinal mucosal barrier injury through the MLCK/pMLC signaling pathway in a piglet model. J Cell Physiol (2021) 236(4):3015–32. 10.1002/jcp.30068 32960454

[32] Chen F Wang H Chen J Liu Y Wen W Li Y. Lactobacillus delbrueckii Ameliorates Intestinal Integrity and Antioxidant Ability in Weaned Piglets after a Lipopolysaccharide Challenge. Oxid Med Cell Longevity (2020) 2020:6028606. 10.1155/2020/6028606 PMC7035547 32104535

[33] Jia L Vianna CR Fukuda M Berglund ED Liu C Tao C. Hepatocyte Toll-like receptor 4 regulates obesity-induced inflammation and insulin resistance. Nat Commun (2014) 5:3878. 10.1038/ncomms4878 24815961 PMC4080408

[34] Ge Y Huang M Yao YM. Autophagy and proinflammatory cytokines: Interactions and clinical implications. Cytokine Growth Factor Rev (2018) 43:38–46. 10.1016/j.cytogfr.2018.07.001 30031632

[35] Albillos A de Gottardi A Rescigno M. The gut-liver axis in liver disease: Pathophysiological basis for therapy. J Hepatol (2020) 72(3):558–77. 10.1016/j.jhep.2019.10.003 31622696

[36] Henao-Mejia J Elinav E Jin C Hao L Mehal WZ Strowig T. Inflammasome-mediated dysbiosis regulates progression of NAFLD and obesity. Nature (2012) 482(7384):179–85. 10.1038/nature10809 PMC3276682 22297845

[37] Boursier J Mueller O Barret M Machado M Fizanne L Araujo-Perez F. The severity of nonalcoholic fatty liver disease is associated with gut dysbiosis and shift in the metabolic function of the gut microbiota. Hepatol (Baltimore Md) (2016) 63(3):764–75. 10.1002/hep.28356 PMC4975935 26600078

[38] Grigor’eva IN. Gallstone Disease, Obesity and the Firmicutes/Bacteroidetes Ratio as a Possible Biomarker of Gut Dysbiosis. J Personalized Med (2020) 11(1):13. 10.3390/jpm11010013 PMC7823692 33375615

[39] Unno T Sakuma M Mitsuhashi S. Effect of dietary supplementation of (-)-epigallocatechin gallate on gut microbiota and biomarkers of colonic fermentation in rats. J Nutr Sci Itaminol (2014) 60(3):213–9. 10.3177/jnsv.60.213 25078378

[40] Shin NR Whon TW Bae JW. Proteobacteria: microbial signature of dysbiosis in gut microbiota. Trends Biotechnol (2015) 33(9):496–503. 10.1016/j.tibtech.2015.06.011 26210164

[41] Frank DN St Amand AL Feldman RA Boedeker EC Harpaz N Pace NR. Molecular-phylogenetic characterization of microbial community imbalances in human inflammatory bowel diseases. Proc Natl Acad Sci USA (2007) 104(34):13780–5. 10.1073/pnas.0706625104 PMC1959459 17699621

[42] Sartor RB. Microbial influences in inflammatory bowel diseases. Gastroenterology (2008) 134(2):577–94. 10.1053/j.gastro.2007.11.059 18242222

[43] Yang J Tang CB Xiao J Du WF Li R. Influences of epigallocatechin gallate and citric acid on Escherichia coli O157:H7 toxin gene expression and virulence-associated stress response. Lett Appl Microbiol (2018) 67(5):435–41. 10.1111/lam.13058 30066955

[44] Spratlin JL Serkova NJ Eckhardt SG. Clinical applications of metabolomics in oncology: a review. Clin Cancer Res Off J Am Assoc Cancer Res (2009) 15(2):431–40. 10.1158/1078-0432.Ccr-08-1059 PMC2676437 19147747

[45] Rivera J Proia RL Olivera A. The alliance of sphingosine-1-phosphate and its receptors in immunity. Nat Rev Immunol (2008) 8(10):753–63. 10.1038/nri2400 PMC2600775 18787560

[46] Cyster JG Schwab SR. Sphingosine-1-phosphate and lymphocyte egress from lymphoid organs. Annu Rev Immunol (2012) 30:69–94. 10.1146/annurev-immunol-020711-075011 22149932

[47] Ueland PM McCann A Midttun Ø Ulvik A. Inflammation, vitamin B6 and related pathways. Mol Aspects Med (2017) 53:10–27. 10.1016/j.mam.2016.08.001 27593095

[48] Kijewski A Witsø IL Iversen H Rønning HT L’Abée-Lund T Wasteson Y. Vitamin K Analogs Influence the Growth and Virulence Potential of Enterohemorrhagic Escherichia coli. Appl Environ Microbiol (2020) 86(24):e00583-20. 10.1128/aem.00583-20 32769190 PMC7688226

[49] McCoin CS Knotts TA Adams SH. Acylcarnitines–old actors auditioning for new roles in metabolic physiology. Nat Rev Endocrinol (2015) 11(10):617–25. 10.1038/nrendo.2015.129 PMC4966159 26303601

