# Supplementary material for: Epigallocatechin Gallate Can Protect Mice From Acute Stress Induced by LPS While Stabilizing Gut Microbes and Serum Metabolites Levels
Source: Front Immunol. 2021 Apr 1;12:640305. doi: 10.3389/fimmu.2021.640305 (PMC8047319; doi:10.3389/fimmu.2021.640305)

**Supplemental Figure 1.** **The protective effect of intragastric administration of different concentrations of EGCG on body weight.** Data are mean ± SD (n = 3) and analyzed by one-way ANOVA. ^a, b, c, d^ mean values with unlike letters were significantly different from each other (*p*<0.05), and the “*P*” value in the figure represents the accurate value of p between the three groups of data, and some of the *p* values between the three groups are too small to be expressed as *p*<0.0001.


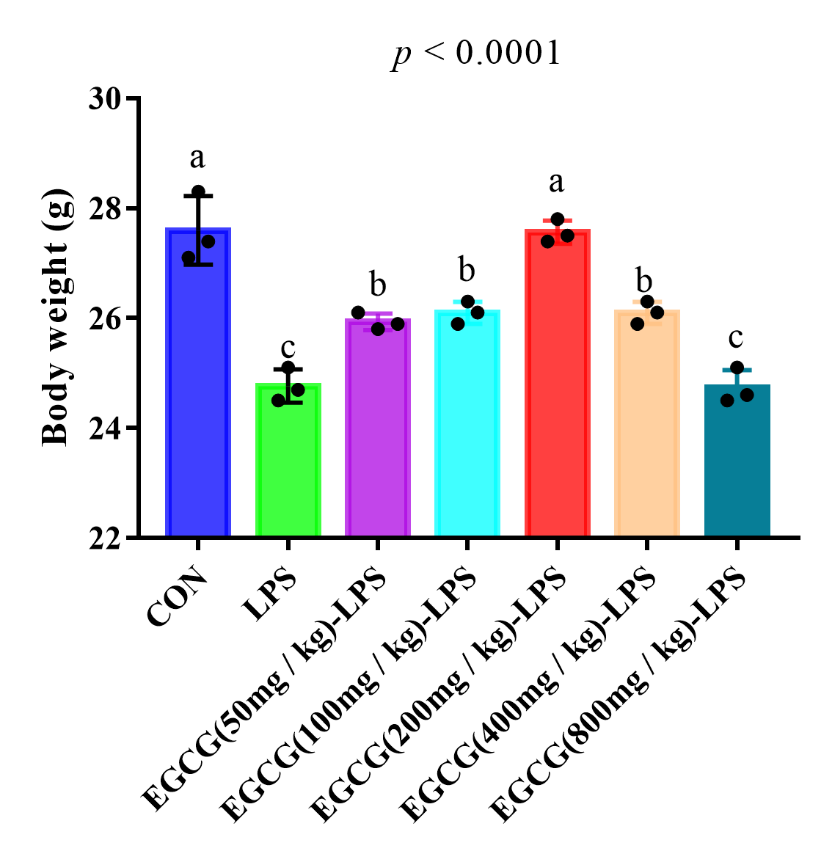


**Supplemental Figure 2. The inhibition of the intragastric administration of different concentrations of EGCG on inflammation in liver tissue.** (A) the concentration of TNF-α in liver tissue; (B) the concentration of IL-1β in liver tissue; (C) the concentration of IL-6 in liver tissue; (D) the concentration of IFN-γ in liver tissue. Data are mean ± SD (n = 3) and analyzed by one-way ANOVA. ^a, b, c, d^ mean values with unlike letters were significantly different from each other (*p*<0.05), and the “P” value in the figure represents the accurate value of p between the three groups of data, and some of the p values between the three groups are too small to be expressed as p<0.0001.


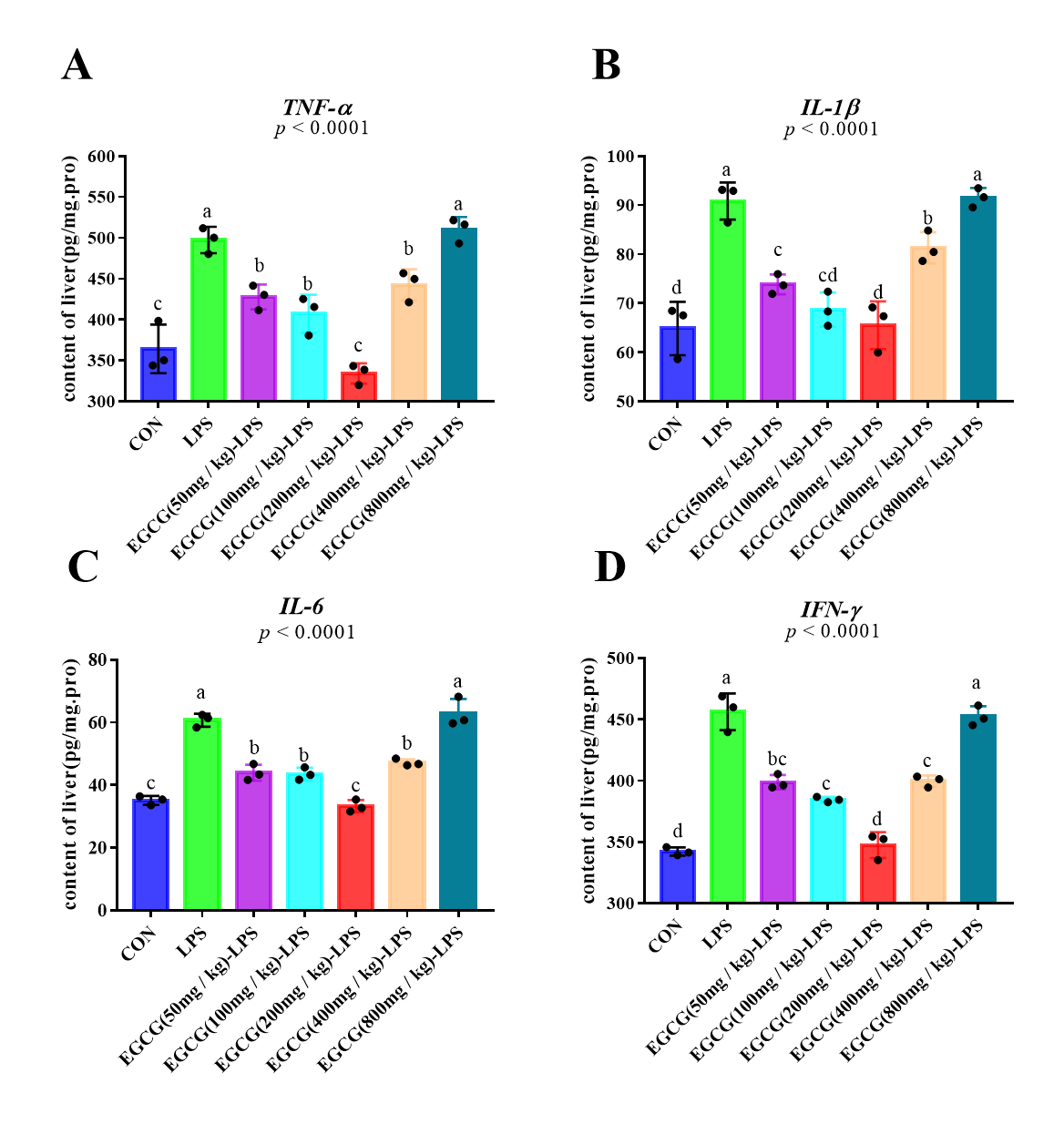

Supplement: Supplementary file 1 [file DataSheet_1.docx]
